# The Treatment of Erysipelas

**Published:** 1892-09

**Authors:** 


THE TREATMENT OF ERYSIPELAS.—(*Therapeutic Gazette*, April, 1892.) In an editorial article, the writer states that in his experience, the treatment which has yielded the best results, consists in washing the part which is affected with a solution of bichloride of mercury in the strength of one to ten thousand, and then thoroughly annointing the skin with an ointment of ichthyol, two drachms to the ounce of lanolin or benzoated lard. This is applied freely, and the part protected by a layer of salicylated cotton and a gauze covering. This treatment, it is claimed, produces a rapid limitation and subsidence of the disease. In addition, however, full doses of the tincture of the chloride of iron should be given and repeated somewhat frequently. In connection with these remarks, the following conclusions reached by Klein, of Warsaw, are given:

1. Ichthyol undoubtedly checks the progress of the disease, either by its reducing action on the tissues, or by its direct action on the micro-organism, or by both actions simultaneously.
2. Ichthyol shortens the mean course of the disease, by about one-half.
3. The treatment is continued from three to four days.
4. The course is considerably milder when ichthyol treatment is employed.

